# Temporary spinal cord stimulation combined with lidocaine patch for postherpetic neuralgia in the elderly: a controlled study

**DOI:** 10.3389/fneur.2025.1529673

**Published:** 2025-01-23

**Authors:** Yun Li, Chunhui Hao, Shengtao Wang, Feng Qiu, Xuli Zhao, Tao Sun

**Affiliations:** ^1^Shandong Provincial Hospital Affiliated to Shandong First Medical University, Jinan, China; ^2^Jinan Second Maternal and Child Health Hospital, Jinan, China

**Keywords:** postherpetic neuralgia, spinal cord stimulation, lidocaine patch, elderly patients, pain management

## Abstract

**Background:**

Postherpetic neuralgia (PHN) is a chronic neuropathic pain condition in elderly patients following herpes zoster infection. Conventional treatments often have inconsistent efficacy and significant side effects. Combining spinal cord stimulation (SCS) with lidocaine patches may enhance pain relief by targeting central and peripheral pain mechanisms.

**Methods:**

This randomized, controlled, single-blind trial enrolled 97 patients aged ≥60 years with PHN lasting ≥6 months. Participants were assigned to SCS with a 5% lidocaine patch (*n* = 49) or SCS with a placebo patch (*n* = 48). Both groups received oral pregabalin. The placebo patch was identical in appearance to ensure blinding. Pain intensity (VAS) and sleep quality (PSQI) were assessed at baseline and on days 1, 3, 7, 30, and 90 post-interventions. Subgroup analyses by age and PHN duration were conducted.

**Results:**

At day 90, the experimental group had greater reductions in VAS scores (1.6 ± 1.1) than the control group (2.7 ± 1.3, *p* < 0.01). Clinically significant pain relief (≥50% VAS reduction) was achieved by 72.3% in the experimental group versus 45.8% in the control group (*p* = 0.038). PSQI scores improved more in the experimental group (5.3 ± 2.1) than in the control group (8.2 ± 2.7, *p* = 0.021). Patients with PHN duration <60 days benefited more from combination therapy. Adverse events were minimal and similar between groups.

**Conclusion:**

Combining SCS with lidocaine patches significantly enhances pain relief and sleep quality in elderly PHN patients compared to SCS alone. Further multicenter studies are recommended to validate these findings and assess long-term outcomes.

**Clinical trial registration:**

https://www.chictr.org.cn/searchprojEN.html, ChiCTR2000039059.

## 1 Introduction

Herpes zoster, resulting from the reactivation of the varicella-zoster virus, predominantly affects older adults due to age-related declines in immune function (1, 2). Among individuals over 60 years old, a significant proportion develop postherpetic neuralgia (PHN), a chronic neuropathic pain condition that persists after the resolution of skin lesions (3, 4). PHN manifests as persistent spontaneous pain, allodynia, and hyperalgesia, significantly impairing quality of life and daily functioning. As the global population ages, the incidence of PHN is increasing, posing a substantial public health challenge (5, 6).

Current management strategies for PHN include oral analgesics, nerve blocks, and topical lidocaine patches; however, their efficacy varies, and side effects are common (7, 8). Oral agents such as gabapentin and pregabalin are frequently prescribed but can cause systemic adverse effects, including dizziness, sedation, and cognitive impairment, which are particularly problematic in elderly patients (9). Nerve blocks may provide temporary relief but require repeated treatments and carry risks of complications such as infection and nerve injury (10). Topical lidocaine offers localized pain relief but may be inadequate for severe or widespread PHN due to limited skin penetration and coverage area (11).

Spinal cord stimulation (SCS) is an established neuromodulation technique that has demonstrated efficacy in managing chronic neuropathic pain, including PHN (12–14). SCS delivers electrical impulses to the dorsal columns of the spinal cord, modulating pain signal transmission and potentially providing significant relief. According to the gate control theory, activation of large-diameter A-*β* fibers inhibits the transmission of nociceptive signals carried by smaller C fibers. Concurrently, lidocaine patches function by blocking voltage-gated sodium channels, reducing ectopic discharges and peripheral sensitization. Combining SCS with lidocaine patches may produce a synergistic effect by targeting both central and peripheral mechanisms of pain, potentially enhancing therapeutic outcomes in elderly patients with PHN (15, 16).

This prospective controlled study aims to evaluate the efficacy and safety of combining temporary SCS with lidocaine patches for treating PHN in the elderly.

## 2 Methods

### 2.1 Study design

This single-center, randomized, controlled, single-blind clinical trial assessed the efficacy and safety of SCS combined with lidocaine patches versus SCS with placebo patches in treating PHN in the elderly. Conducted from January 2022 to December 2023, the study received approval from the Institutional Review Board (IRB) of the participating hospital. Written informed consent was obtained from all participants prior to enrollment. The trial was registered with the Chinese Clinical Trial Registry (number: ChiCTR2000039059).

### 2.2 Participants

We included adults aged 60 years or older who experienced postherpetic neuralgia (PHN), defined as pain persisting for at least 6 months following the healing of herpes zoster lesions. All participants had PHN localized to the thoracic or lumbar regions, as confirmed through clinical examination and patient reports. At the time of enrollment, participants reported a pain level of 4 or higher on the Visual Analog Scale (VAS). Additionally, all participants had failed to achieve adequate pain relief with standard pharmacological treatments, including oral analgesics such as pregabalin and gabapentin, despite optimal dosing and adherence for a minimum of 3 months.

Individuals were excluded if they had a history of blood clotting disorders or active bleeding, severe psychiatric conditions that could hinder their participation, or known allergies to lidocaine or its components. We also excluded those with conditions that made spinal cord stimulation unsuitable or who had received nerve-modulating therapies within the past year.

**Figure 1:**
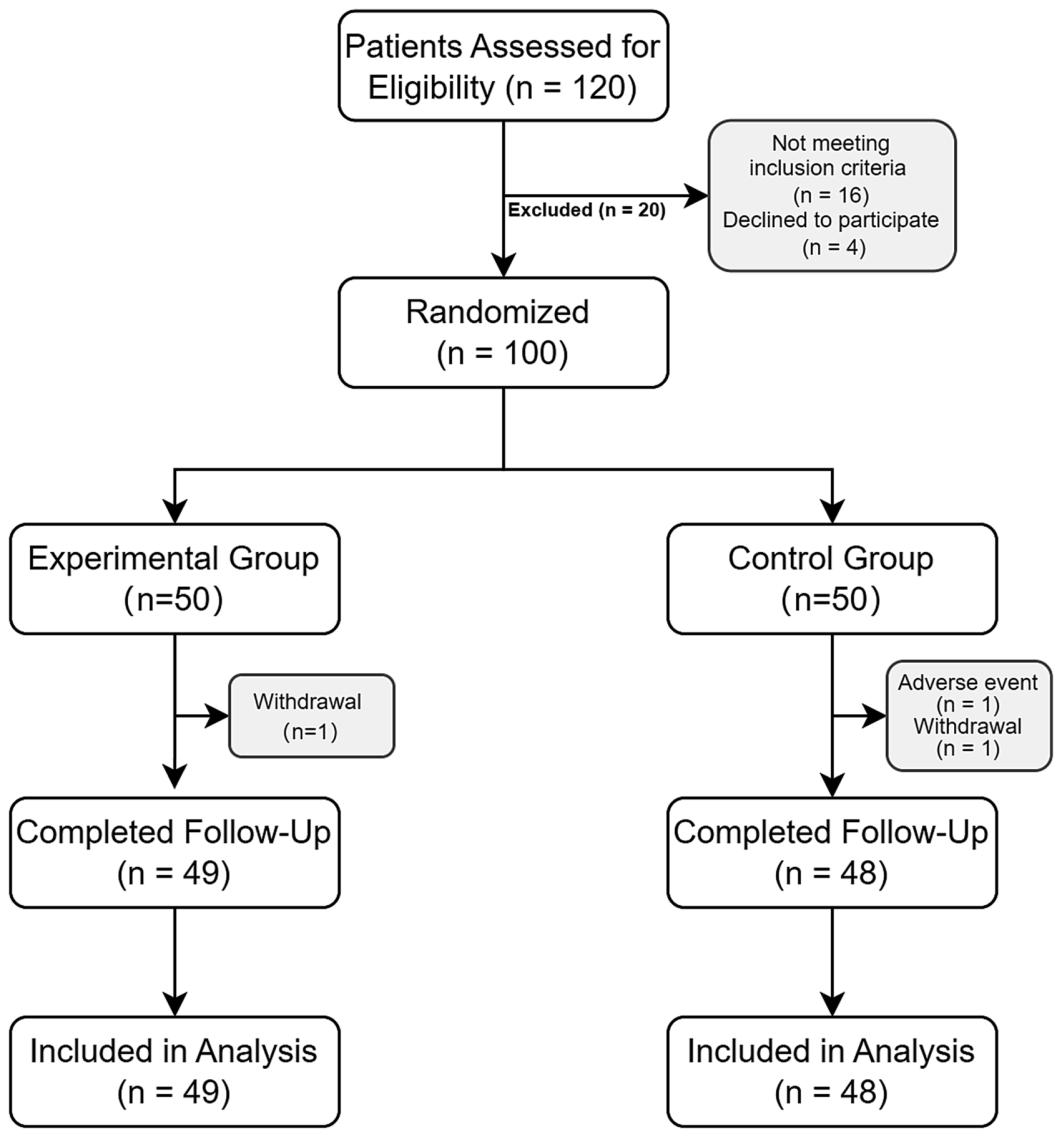
Flowchart of the study. Represents the CONSORT flow diagram of participants.

Before undergoing spinal cord stimulation surgery, all patients were evaluated by a psychologist or psychiatrist specializing in pain management. Based on this assessment, patients with active psychiatric disorders or ongoing substance abuse—including alcohol dependence—were excluded. All participants were capable of providing written informed consent and agreed to comply with the study procedures. Figure 1 presents the flow diagram of participant enrollment and allocation.

### 2.3 Randomization and blinding

Participants meeting the inclusion criteria were randomly assigned in a 1:1 ratio to either the treatment group (SCS with lidocaine patch) or the control group (SCS with placebo patch). Randomization was performed using a computer-generated sequence and managed by an independent research coordinator not involved in clinical evaluations or interventions. The study was single-blind; participants were unaware of their group assignments, while clinicians administering treatments were aware due to the nature of the interventions.

### 2.4 Interventions

#### 2.4.1 Spinal cord stimulation

Prior to surgery, all patients received comprehensive health education to ensure understanding and compliance with postoperative care protocols. SCS procedures were performed under local anesthesia. Patients were positioned in the prone position and guided using fluoroscopic X-ray imaging. Peripheral venous access was established, and vital signs were continuously monitored throughout the procedure.

The puncture site was selected based on the dermatomal distribution corresponding to the patient’s area of pain. Under fluoroscopic guidance, a 14-gage epidural needle was percutaneously inserted into the epidural space. An 8-contact epidural stimulation lead was then advanced through the needle into the dorsal epidural space, positioned at the vertebral level corresponding to the patient’s pain distribution (17). The spinal cord stimulation devices used in this study were manufactured by Medtronic (Model 977D260).

The lead extension was connected to an external pulse generator for intraoperative stimulation testing. Stimulation parameters were set with pulse widths of 200–220 μs, frequencies of 40–80 Hz, and amplitudes adjusted between 0.5 and 5 mA. The stimulation intensity was gradually increased while adjusting the lead position based on patient feedback. Adjustments continued until the patient reported paresthesia encompassing the entire painful area, indicating optimal coverage.

Once adequate stimulation coverage was achieved, the needle was withdrawn. The lead was secured at the skin entry point using tension-relieving sutures, and a sterile dressing was applied to the puncture site. Patients were advised to limit strenuous activity for at least 2 weeks post-procedure to prevent lead displacement.

#### 2.4.2 Postoperative management

Postoperatively, patients were advised to limit activity for 24 h following the procedure to prevent electrode displacement. External stimulation continued for 14 days post-procedure, during which stimulation parameters were adjusted daily based on pain scores and coverage area. Regular wound care and infection monitoring were conducted, and routine radiographic evaluations confirmed electrode position.

#### 2.4.3 Treatment protocol

- Intervention Group: A 5% lidocaine gel patch (Each patch contains 14 g of ointment and 700 mg of lidocaine) was applied daily to the affected dermatomes for up to 12 h per application. If multiple painful areas were reported, the patches were applied to cover the region with the highest pain intensity, as identified by the patient during baseline assessment.
- Control Group: An identical protocol using placebo patches was implemented.

### 2.5 Outcome measures

#### 2.5.1 Changes in pain intensity

Assessed using the VAS at baseline and on days 1, 3, 7, 30, and 90 post-interventions. The VAS is a 10-point scale ranging from 0 (no pain) to 10 (worst possible pain).

#### 2.5.2 Sleep quality

Evaluated using the Pittsburgh Sleep Quality Index (PSQI) at the same time points. The PSQI comprises 19 self-rated items, generating a total score ranging from 0 to 21, with higher scores indicating poorer sleep quality.

#### 2.5.3 Adverse events

Monitored for the frequency and nature of adverse events, including skin reactions at the patch application site, electrode migration, and other procedure-related complications.

### 2.6 Sample size calculation

The sample size was calculated to detect a minimum clinically important difference of 1.0 in VAS scores between groups at day 90, with a standard deviation of 1.5, a power of 80%, and a two-sided alpha level of 0.05. This resulted in a required sample size of 45 patients per group, accounting for a 10% dropout rate.

### 2.7 Statistical analysis

Statistical analyses were conducted using SPSS version 25.0 (IBM Corp., Armonk, NY, United States). Continuous variables are presented as mean ± standard deviation (SD) or median with interquartile range (IQR) based on normality assessed using the Shapiro–Wilk test. Categorical variables are expressed as counts and percentages. Independent samples t-tests were utilized for normally distributed continuous data, and the Mann–Whitney U test was employed for non-normally distributed data. Categorical variables were analyzed using Chi-square tests or Fisher’s exact tests, as appropriate. Changes in pain intensity and sleep quality over time between the two groups were evaluated using repeated measures analysis of variance (ANOVA). If the assumption of sphericity was violated, the Greenhouse–Geisser correction was applied. A *p*-value of less than 0.05 was considered statistically significant. Data visualization was performed using R (R Foundation for Statistical Computing, Vienna, Austria).

### 2.8 Ethical considerations

This study was conducted in accordance with the Declaration of Helsinki and was approved by the Institutional Review Board (IRB) of Shandong Provincial Hospital (Approval Number: 2022-slyy-217). Written informed consent was obtained from all participants prior to enrollment.

## 3 Results

### 3.1 Baseline characteristics

A total of 100 patients were initially enrolled in the study. After excluding three patients due to incomplete follow-up data, 97 patients were analyzed—49 in the experimental group and 48 in the control group. Table 1 summarizes their baseline characteristics. There were no significant differences between the groups in terms of age, gender, duration of PHN, baseline VAS scores, or comorbidities such as hypertension and diabetes (all *p*-values >0.05).

**Table 1:** Baseline characteristics.

Characteristic	Experimental group (*n* = 49)	Control group (*n* = 48)	*p*- value
Age (years)	68.5 ± 7.3	69.1 ± 6.8	0.56
Gender (Male/Female)	24/25	23/25	0.87
Duration of PHN (days)	45.3 ± 12.1	47.6 ± 11.9	0.31
Baseline VAS Score	7.2 ± 1.4	7.1 ± 1.3	0.69
Smoking History (Yes/No)	15/34	16/32	0.78
Hypertension (Yes/No)	22/27	21/27	0.90
Diabetes (Yes/No)	8/41	7/41	0.85

Data are presented as mean ± standard deviation or number.

### 3.2 Pain relief

**Figure 2:**
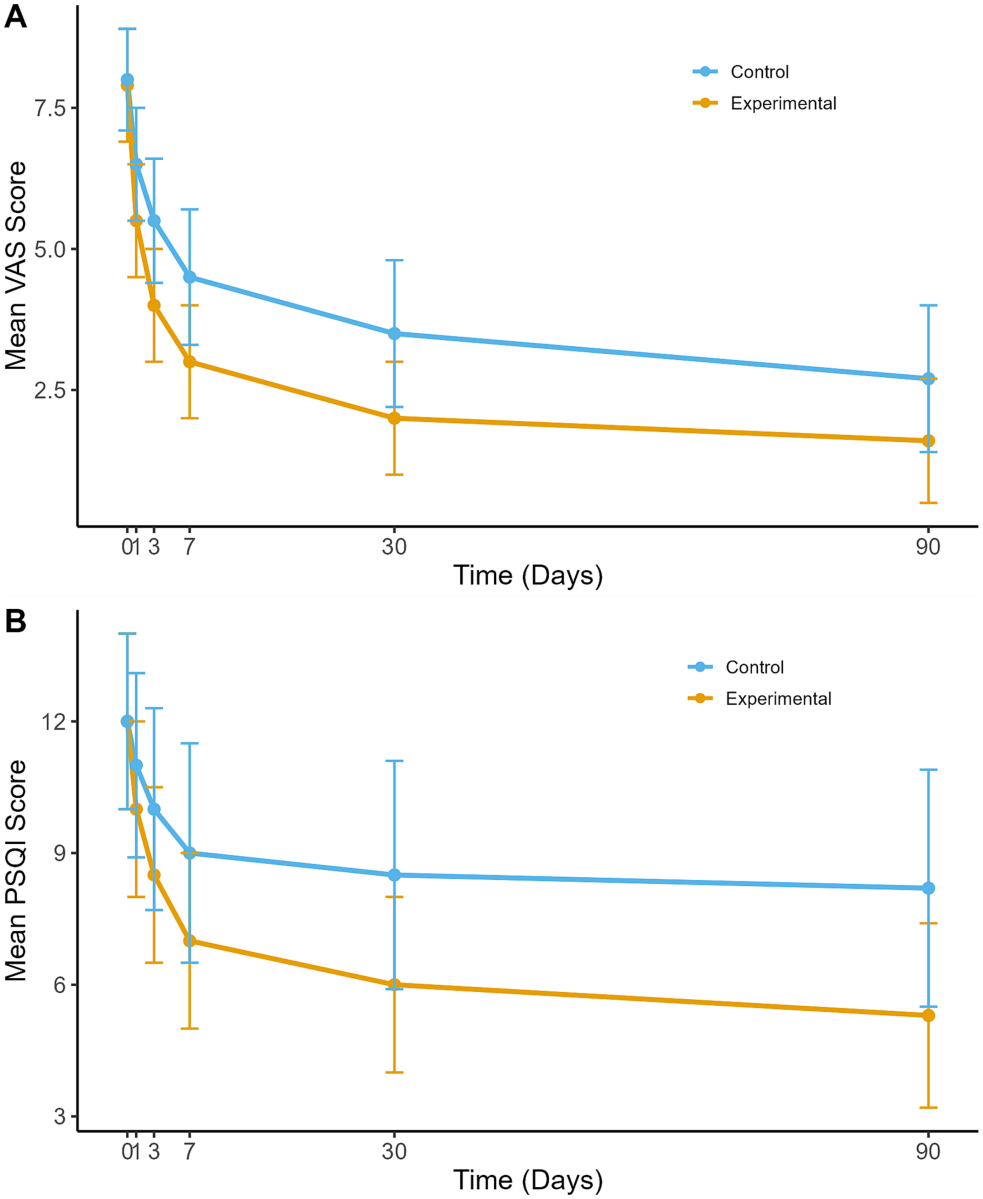
Mean VAS and PSQI scores over time. **(A)** Mean VAS scores over time for the experimental and control groups. **(B)** Mean PSQI scores over time for the experimental and control groups. Error bars represent standard deviations.

Pain intensity, measured by the VAS, decreased significantly in both groups at all evaluated time points compared to baseline (all *p*- values <0.01). However, the reductions were more pronounced in the experimental group. Significant between-group differences were observed, especially at days 3, 7, 30, and 90 post-treatments (Figure 2A). At day 90, the mean VAS score was 1.6 ± 1.1 in the experimental group and 2.7 ± 1.3 in the control group, resulting in a mean difference of-1.1 (95% CI: −1.5 to-0.7; *p* < 0.01).

Clinically significant pain relief, defined as a ≥ 50% reduction in VAS scores, was achieved by 72.3% of patients in the experimental group compared to 45.8% in the control group at day 90 (*p* = 0.038).

### 3.3 Sleep quality

Both groups showed significant improvements in sleep quality over time, as assessed by the PSQI (all *p*-values <0.01). The experimental group experienced more substantial improvements than the control group at all post-treatment assessments (Figure 2B). By day 90, the mean PSQI score was 5.3 ± 2.1 in the experimental group versus 8.2 ± 2.7 in the control group, indicating better sleep quality in the experimental group (mean difference: -2.9; 95% CI: −4.1 to-1.7; *p* = 0.021).

### 3.4 Subgroup analysis

To explore factors influencing treatment efficacy, subgroup analyses were conducted based on age and duration of PHN symptoms. In subgroup analyses based on age and duration of PHN symptoms, we assessed potential demographic differences to rule out confounding factors. Statistical analysis revealed no significant demographic differences between the experimental and control groups within each age and PHN duration subgroup (*p* > 0.05).

#### 3.4.1 Age groups

Patients were stratified into two age groups: 60–69 years and ≥ 70 years. In both age groups, the experimental group showed significantly greater reductions in VAS scores and improvements in PSQI scores compared to the control group.

- 60–69 Years age group: At day 90, the mean VAS score in the experimental group decreased from 7.9 ± 1.0 to 1.5 ± 1.0, while the control group decreased from 8.0 ± 0.9 to 2.6 ± 1.2 (mean difference: -1.1; 95% CI: −1.8 to-0.4; *p* = 0.002). The mean PSQI score reduction was 5.5 ± 2.0 in the experimental group compared to 3.2 ± 2.3 in the control group (*p* = 0.020).
- ≥70 Years age group: At day 90, the experimental group’s mean VAS score decreased from 8.1 ± 1.1 to 1.7 ± 1.2, compared to a reduction from 8.0 ± 1.0 to 2.8 ± 1.3 in the control group (mean difference: -1.1; 95% CI: −1.9 to-0.3; *p* = 0.005). The mean PSQI score reduction was 4.9 ± 2.1 in the experimental group versus 2.9 ± 2.4 in the control group (*p* = 0.029).

**Figure 3:**
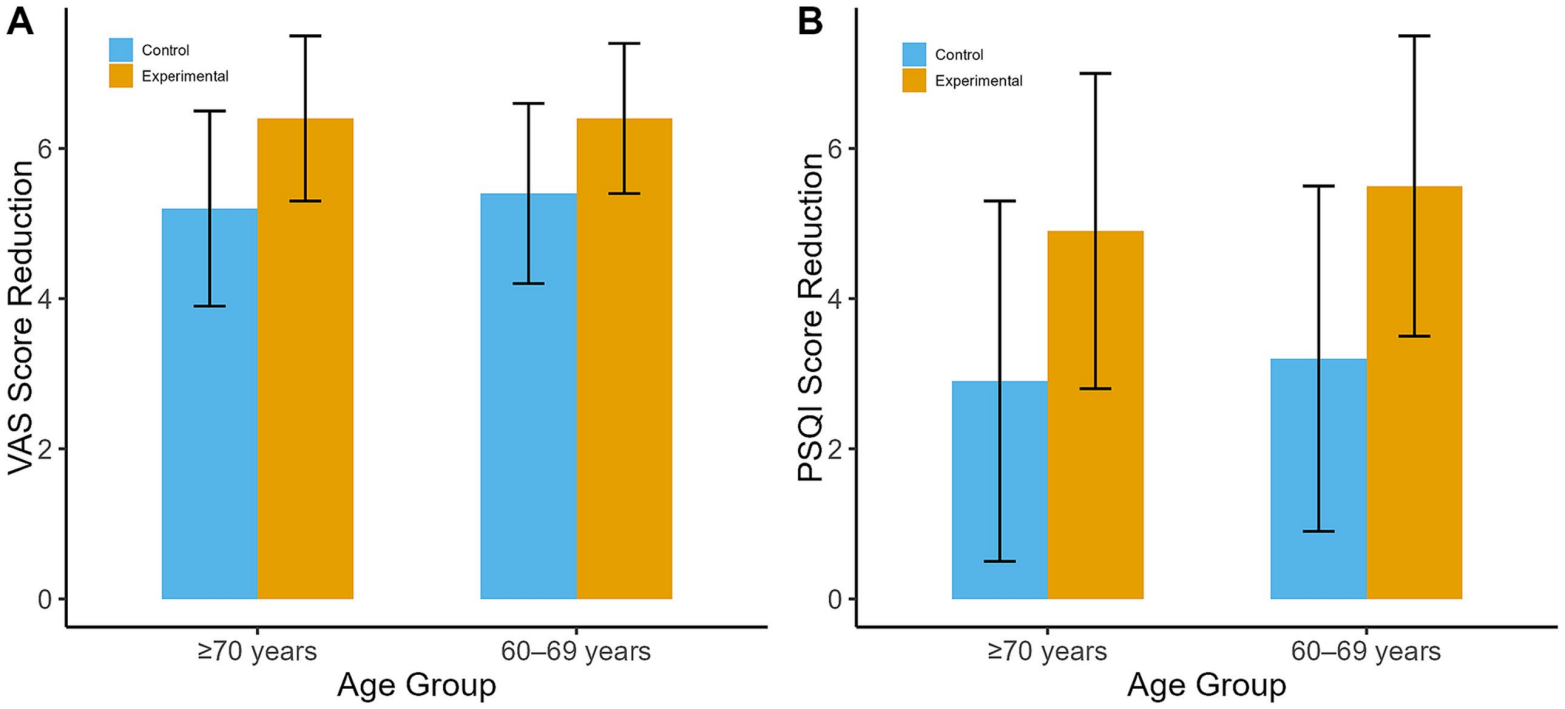
Subgroup analysis by age group. **(A)** VAS score reduction at day 90 by age group. **(B)** PSQI score reduction at day 90 by age group.

These findings are illustrated in Figure 3.

While both age groups benefited from the combination therapy, statistical analysis revealed a trend toward interaction between age and treatment effect for pain reduction (*p* = 0.08) and sleep quality (*p* = 0.12), though these did not reach statistical significance. This suggests that age may have a minimal effect on treatment efficacy, but further research with a larger sample size is needed to confirm these findings.

#### 3.4.2 PHN duration

Patients were also categorized based on the duration of PHN symptoms at enrollment: less than 60 days and 60 days or more.

- Duration < 60 days: At day 90, the experimental group experienced a mean VAS score reduction of 5.0 ± 1.2, compared to 3.7 ± 1.4 in the control group (mean difference: -1.3; 95% CI: −2.1 to-0.5; *p* = 0.001). The mean PSQI score reduction was 5.7 ± 1.9 in the experimental group versus 3.4 ± 2.2 in the control group (*p* < 0.01).
- Duration ≥ 60 days: The experimental group showed better outcomes than the control group, but the differences were less pronounced. At day 90, the mean VAS score reduction was 4.2 ± 1.3 in the experimental group versus 3.4 ± 1.5 in the control group (mean difference: -0.8; 95% CI: −1.6 to 0.0; *p* = 0.05). The mean PSQI score reduction was 4.4 ± 2.0 in the experimental group compared to 2.7 ± 2.3 in the control group (*p* = 0.004).

**Figure 4:**
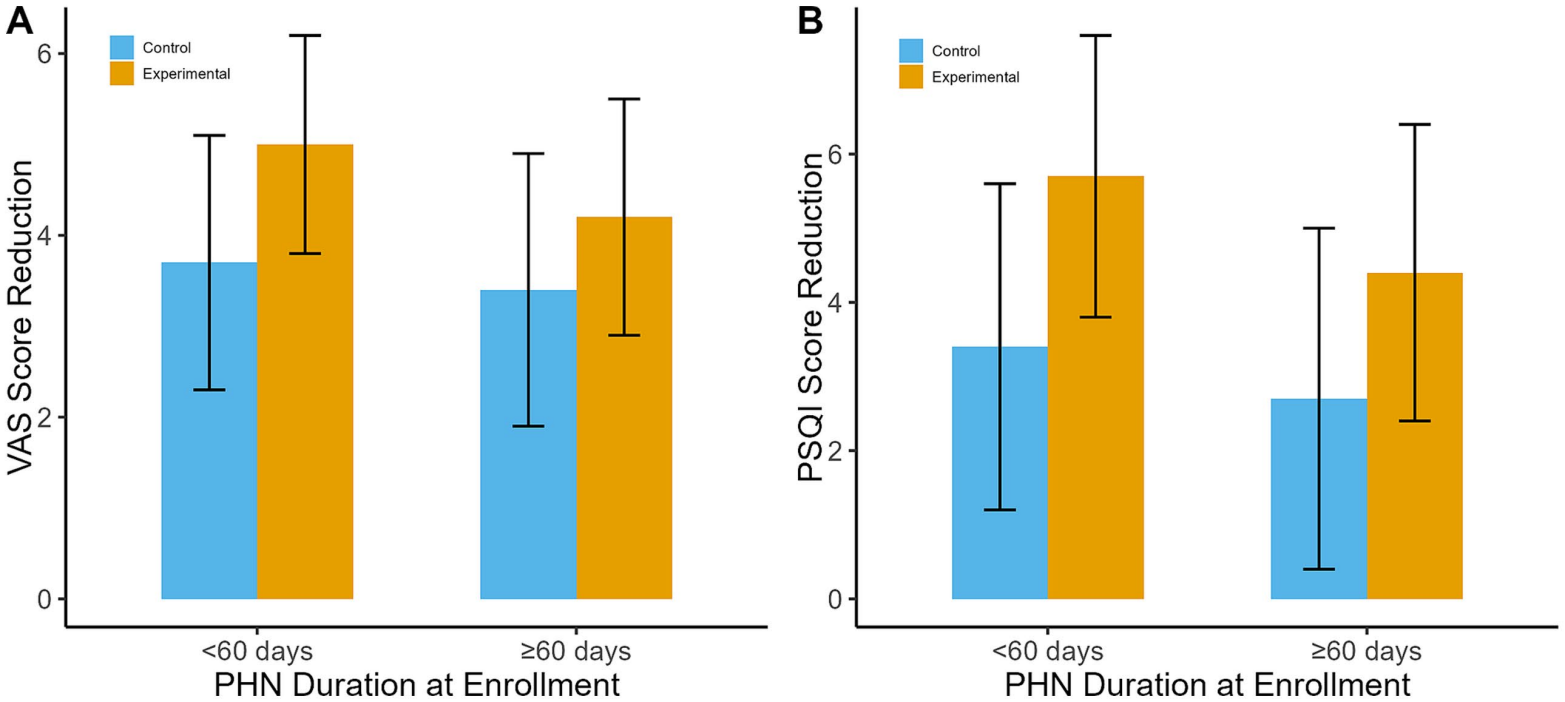
Subgroup analysis by PHN duration. **(A)** VAS score reduction at day 90 by PHN duration. **(B)** PSQI score reduction at day 90 by PHN duration.

These results are presented in Figure 4.

Statistical analysis indicated a significant interaction between PHN duration and treatment effect for pain reduction (*p* = 0.03), suggesting that patients with a shorter duration of PHN may benefit more from the combination therapy. This highlights the potential importance of early intervention.

### 3.5 Safety and complications

Adverse events were minimal and comparable between the groups. Mild skin reactions at the patch application site were reported in two patients in the experimental group and one patient in the control group (*p* = 0.58), all of which resolved without intervention. One patient in the experimental group experienced mild sedation, which also resolved spontaneously. There were no instances of electrode migration, infection, or other serious complications, indicating that the combination therapy was well-tolerated.

## 4 Discussion

This study evaluated the efficacy and safety of combining temporary SCS with lidocaine patches for treating PHN in elderly patients. Our results demonstrated that the combination therapy significantly reduces pain intensity compared to SCS alone, with the most notable effects observed at 90 days post-treatment. At day 90, the experimental group reported a mean VAS score of 1.6 ± 1.1, compared to 2.7 ± 1.3 in the control group. Additionally, improvements in sleep quality, as measured by the PSQI, were more substantial in the experimental group, suggesting broader benefits to patient well-being. These findings substantiate a multimodal treatment approach for PHN, a condition notoriously difficult to manage with standard therapies (18).

Subgroup analyses revealed that the combination of SCS with lidocaine patches is effective across different age groups among elderly patients with PHN. While patients aged 60–69 years exhibited slightly greater improvements, age did not significantly alter treatment efficacy. Furthermore, patients with a shorter duration of PHN symptoms (<60 days) experienced more substantial benefits from the combination therapy compared to those with a longer duration of symptoms. These findings emphasize the potential benefits of prompt initiation of combination therapy in elderly patients with PHN.

The clinical significance of our findings lies in the potential to enhance the management of PHN, a condition that profoundly affects patient quality of life. Theoretically, this study enriches our understanding of pain modulation through the dual mechanisms of SCS and lidocaine patches. Specifically, SCS likely inhibits pain transmission by activating large-diameter A-*β* fibers (19–21), while lidocaine patches target peripheral sensitization by blocking voltage-gated sodium channels (22). This combination may effectively address both central and peripheral aspects of neuropathic pain, offering a comprehensive pain management strategy.

In addition to SCS and lidocaine patches, several other treatment modalities have been explored for the management of PHN. Radiofrequency ablation targets specific nerves to disrupt pain signal transmission and has demonstrated efficacy in reducing pain intensity, particularly in localized PHN (23). However, its analgesic effects are often temporary, necessitating repeated procedures to maintain pain relief. Botulinum toxin injections have emerged as a promising option by inhibiting the release of acetylcholine at neuromuscular junctions, thereby reducing muscle spasms and modulating pain pathways (24). Nerve blocks, including epidural and peripheral nerve blocks, provide temporary pain relief by interrupting nociceptive signal transmission and are particularly useful during acute exacerbations of PHN (25). Similarly, capsaicin 8% patches function by desensitizing sensory neurons through the depletion of substance P, thereby diminishing pain signals. While studies have shown their effectiveness in lowering pain scores, the initial burning sensation experienced by some patients can limit their tolerability (26).

From a practical standpoint, combined SCS and lidocaine patch therapy could be a valuable addition to the treatment options for elderly patients with PHN, particularly those inadequately managed by monotherapy (22). This approach may also reduce dependence on systemic medications, which are often associated with significant side effects in elderly populations. To implement these findings clinically, it is recommended to integrate this combined therapy into existing PHN management protocols in geriatric care and to develop training programs for healthcare providers to enhance awareness and adoption of this evidence-supported treatment modality (27, 28).

Despite the encouraging findings, certain limitations should be acknowledged. Firstly, the sample size and single-center design may affect the generalizability of the results. Additionally, the observation period of up to 90 days is relatively short, limiting our ability to assess the long-term efficacy and safety of the combined SCS and lidocaine patch therapy. Future studies could benefit from larger, multicenter trials with extended follow-up periods to comprehensively validate these findings. Furthermore, integrating psychological therapies, such as cognitive-behavioral therapy (CBT), with the combined SCS and lidocaine patch treatment may further enhance patient outcomes by addressing the psychological aspects of chronic pain management (29, 30).

## 5 Conclusion

In conclusion, our study provides compelling evidence that the combined use of SCS and lidocaine patches is an effective treatment strategy for managing PHN in elderly patients. These findings underscore the potential of a multimodal approach to significantly enhance clinical outcomes and improve patient well-being. However, to fully validate these benefits and facilitate the widespread integration of this combination therapy into standard clinical practice, broader and longer-term studies are necessary.

## Data Availability

The original contributions presented in the study are included in the article/supplementary material, further inquiries can be directed to the corresponding author/s.
